# Stem Cell Control, Oscillations, and Tissue Regeneration in Spatial and Non-Spatial Models

**DOI:** 10.3389/fonc.2013.00082

**Published:** 2013-04-15

**Authors:** Ignacio A. Rodriguez-Brenes, Dominik Wodarz, Natalia L. Komarova

**Affiliations:** ^1^Department of Mathematics, University of California IrvineIrvine, CA, USA; ^2^Department of Ecology and Evolutionary Biology, University of California IrvineIrvine, CA, USA

**Keywords:** tissue regeneration, cell linage control, tissue stability, mathematical models, cancer

## Abstract

Normal human tissue is organized into cell lineages, in which the highly differentiated mature cells that perform tissue functions are the end product of an orderly tissue-specific sequence of divisions that start with stem cells or progenitor cells. Tissue homeostasis and effective regeneration after injuries requires tight regulation of these cell lineages and feedback loops play a fundamental role in this regard. In particular, signals secreted from differentiated cells that inhibit stem cell division and stem cell self-renewal are important in establishing control. In this article we study in detail the cell dynamics that arise from this control mechanism. These dynamics are fundamental to our understanding of cancer, given that tumor initiation requires an escape from tissue regulation. Knowledge on the processes of cellular control can provide insights into the pathways that lead to deregulation and consequently cancer development.

## Introduction

There is growing evidence that a subset of cancer cells possesses characteristics typically associated with stem cells (Reya et al., 2001; Wang et al., 2010). These so called cancer stem cells share with normal stem cells the capability to give rise to all cell types of a given lineage (Bonnet and Dick, 1997; Passegué et al., 2003). Like normal stem cells, they also have a large proliferative potential being the only cancer cells capable of repopulating a tumor and initiating metastasis (Al-Hajj et al., 2003; Clevers, 2011). In light of these findings it is crucial to understand how stem cells are regulated as part of a cell lineage in normal tissue.

In normal tissues, cell lineages are highly regulated to promote the rapid regeneration after an injury and to maintain tissue homeostasis under normal conditions. In particular when it comes to the regulation of stem cells two types of feedbacks have been proposed: long-range and short-range (Arino and Kimmel, 1986). The long-range feedbacks should respond to the loss of mature cells during an injury, while the short-range feedbacks would act in an autocrine fashion in stem cells (Andersen and Mackey, 2001; Bernard et al., 2003). In this article we focus on long-range feedback acting through signals emitted by differentiated cells that inhibit stem cell division and self-replication. This type of regulation has been biologically observed in numerous tissues including muscle, liver, bone, and the nervous and hematopoietic systems (McPherron et al., 1997; Daluiski et al., 2001; Yamasaki et al., 2003; Elgjo and Reichelt, 2004; Tzeng et al., 2011), and has lead to the development of a significant number of mathematical models (see e.g., Ganguly and Puri, 2006; Lander et al., 2009; Marciniak-Czochra et al., 2009; Chou et al., 2010; Bocharov et al., 2011; Zhang et al., 2012).

Tumor initiation requires an escape from the control mechanisms just described and indeed, there is significant experimental evidence to support this assertion (Lim et al., 2000; Massagué, 2000; Woodford-Richens et al., 2001; Piccirillo et al., 2006; Lee et al., 2008). This underscores the importance of tissue regulation for cancer biology. In the next sections we will analyze the cell dynamics resulting from this regulatory mechanism, first in the context of general feedback functions and then using Hill equations in spatial and non-spatial settings.

Our work adds to a growing body of modeling literature that studies cell lineage dynamics and regulation. Conceptual issues for the study of stem cells are identified in Loeffler and Roeder (2002). Discrete and continuous models relevant to carcinogenesis, and particularly colon cancer, include (Tomlinson and Bodmer, 1995; Yatabe et al., 2001; Agur et al., 2002; Hardy and Stark, 2002; d’Onofrio and Tomlinson, 2007; Johnston et al., 2007; Boman et al., 2008). There are also numerous stem cell models in the context of the hematopoietic system (see e.g., Colijn and Mackey, 2005; Michor et al., 2005; Adimy et al., 2006; Glauche et al., 2007; Ashkenazi et al., 2008). In this paper we combine elements of stochastic and deterministic modeling and consider both mass action and spatial systems. The models identify parameters important for tissue stability and growth and offer a useful tool to study both healthy and cancerous hierarchical populations.

The stability and dynamics of multistage cell lineage models is an active topic of research. In Nakata et al. (2012), the authors systematically analyze the stability of a two and three compartment model where the regulation of proliferation rates is modeled using Hill functions equation (9). A similar model where feedback regulation acts instead on the probability of self-renewal is studied in Lo et al. (2009); here the stability analysis is performed first using a general feedback function for a two compartment model, and then using the feedback function equation (9) for a three compartment model. In Stiehl and Marciniak-Czochra (2011) the authors characterize the structure of stationary solutions of a n-compartment model with feedback on the self-renewal probability of cells. The characterization is performed for a general form of the regulation function and for the special case that uses the functional form in equation (9).

In this article we study the cell dynamics of a two compartment model, which includes feedback regulation in both the division rate and the self-renewal probability of cells. According to the model feedback on the self-renewal probability of stem cells is by itself sufficient to establish control. However if feedback on the division rate is not present, the recovery after an injury may lead to significant damped oscillations in the path back to equilibrium, which can result in the stochastic extinction of the cell population. Moreover, this oscillatory behavior is more pronounced when the stem cell load represents only a small fraction of the entire cell population. If this is the case, oscillations may still be avoided, but it comes at the price of slowing down the speed at which the system is able to recover after an injury. Spatial interactions and the addition of feedback inhibition on the cell division rate reduce the amplitude of oscillations and contributes to the robustness of the system. Feedback inhibition on the division rate also increases the speed of tissue regeneration promoting altogether faster and more stable recoveries from perturbed states.

## Results

### Cellular control

**Figure 1:**
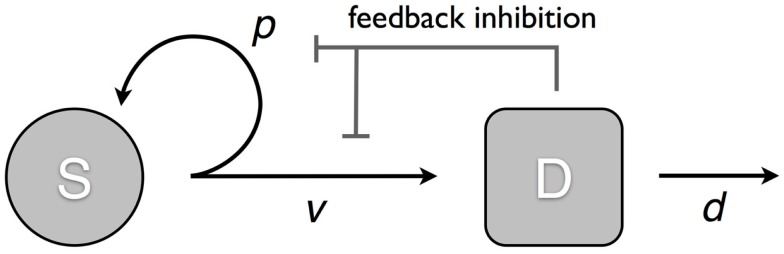
**Model of tissue regulation with feedback loops**. *S* represents the stem cell population and *D* the differentiated cell population. Stem cells divide at a rate *v*; this results in either two daughter stem cells with probability *p*; or two differentiated cells with probability 1* − p*. Differentiated cells die at rate *d*. The rate of cell division and the probability of self-renewal are decreasing functions of the number of differentiated cells [equation (1)].

We consider a model that takes into account two cell populations: stem cells, *S*, which have unlimited reproductive potential, and differentiated cells *D*, that eventually die out (this includes all cell populations with limited reproductive potential, such as transit cells). Stem cells divide at a rate *v*; this results in either two daughter stem cells with probability *p* or two differentiated cells with probability 1 − *p*. Differentiated cells die at rate *d*. The system is controlled through two negative feedback loops. Differentiated cells secrete factors that: (1) inhibit stem cell division, and (2) suppress self-renewal in stem cells (Figure 1). Hence, the self-renewal probability and division rate (*p*(*D*) and *v*(*D*)) are strictly decreasing functions of the number of differentiated cells *D*. The ordinary differential equation (ode) model is given by:

(1) $$\begin{array}{l} Ṡ=\left(2p\left(D\right)-1\right)\upsilon \left(D\right)S\\ Ḋ=2\left(1-p\left(D\right)\right)v\left(D\right)S-dD \end{array}$$

In addition to the symmetric stem cell divisions explicitly modeled in equation (1) asymmetric division in stem cells is also well documented (Clevers, 2005; Simons and Clevers, 2011). The extent to which these types of divisions occur in different tissues has important biological consequences and is the subject of considerable research efforts (Wu et al., 2007; Neumüller and Knoblich, 2009). However with regards to model (1), it is shown in Rodriguez-Brenes et al. (2011) (Supplementary Information) that the explicit introduction of asymmetric stem cell divisions leads to an equivalent mathematical formulation and does not alter any of the results.

From the expression for Ṡ, we note that *p*(0) > 0.5 is a necessary condition to avoid the system from always going to the trivial steady solution (*S*, *D*) = (0, 0). Also only feedback on the self-renewal probability *p* – unlike the feedback on *v* – is able to change the signs of *Ṡ* or *Ḋ*, which suggests that by itself feedback inhibition on *p* is sufficient to maintain control. We are interested in finding out how this negative regulation affects the cell population at homeostasis and during recovery after an injury. We begin by looking at the steady states $Ŝ\;\text{ and }\;\hat{D}$ and $\hat{D}$ which are defined by the following equations:

(2) $$p\left(\hat{D}\right)=1∕2\;\text{\& }\;Ŝ=d∕v\left(\hat{D}\right)\hat{D}$$

Hence, we find that the equilibrium number of differentiated cells $\hat{D}$ depends only on the self-renewal probability *p*(*D*). The equilibrium fraction of stem cells $Ŝ∕\left(Ŝ+\hat{D}\right)$ depends only on the ratio $d∕v\left(\hat{D}\right).$ In order to understand better the recovery of the system after a perturbation we look at the eigenvalues of the Jacobian matrix evaluated at $\left(Ŝ,\hat{D}\right)$:

(3) $$J=\left[\;\begin{array}{cc} 0 & 2dp^{\prime }\left(\hat{D}\right)\hat{D}\\ v\left(\hat{D}\right) & -d\left(2p^{\prime }\left(\hat{D}\right)\hat{D}+1\right) \end{array}\right]$$

Let us write $b=\left(2p^{\prime }\left(\hat{D}\right)\hat{D}+1\right)$ and $\hat{v}=v\left(\hat{D}\right).$ Then the eigenvalues are given by:

(4) $$\lambda_{1},\lambda_{2}=\frac{-db\pm \sqrt{d^{2}b^{2}+4d\left(b-1\right)\hat{v}}}{2}$$

The model described by equation (1) is an autonomous system of ordinary differential equations; therefore in a vicinity of the steady state point $\left(Ŝ,\;\hat{D}\right)$ the behavior of the system can be inferred by looking at the eigenvalues of the Jacobian. If we want the equilibrium values to be asymptotically stable, then the real part of the eigenvalues must be negative, which occurs if and only if *b* > 0. Conversely if *b* < 0, the equilibrium is unstable. If *b* = 0 (purely imaginary eigenvalues), the behavior of the system can not be inferred from equation (1) for general functions *v*(*D*) and *p*(*D*). In this case a Hopf bifurcation might be possible. However the bifurcation analysis would depend on the specific choice of the regulation functions.

The sign of the discriminant in equation (4) gives us further information into how the trajectories approach the steady state value. If the discriminant is negative then oscillations are expected as the cell population approaches equilibrium. Let us see how this observation relates to the equilibrium fraction of stem cells in the population. As we noted earlier this fraction is entirely determined by the ratio $∊\equiv d∕\hat{v}$. If we want to avoid oscillations then dividing the discriminant by $d\hat{v}$ we find that the following inequality must hold:

(5) $$∊b^{2}+4b-4\geq 0$$

Since $b=1+2p^{\prime }\left(\hat{D}\right)\hat{D}$ we have *b* < 1 and if a stable steady exists we then have 0 < *b* < 1. Hence the inequality in equation (5) implies that:

(6) $$b\geq \frac{-2+2\sqrt{1+∊}}{∊}$$

Stem cells typically represent a small fraction of the entire cell population which in terms of the ratio ∊ equals ∊/(1 + ∊). As ∊ approaches zero we find:

(7) $$\underset{∊\to 0}{\lim}\frac{-2+2\sqrt{1+∊}}{∊}=1$$

Given the inequality found in equation (6) and the fact that *b* < 1 we find that as the equilibrium fraction of stem cells approaches zero, *b* approaches one. For the eigenvalues we then have:

(8) $$\underset{b\to 1^{-}}{\lim}\frac{-db\pm \sqrt{d^{2}b^{2}+4d(b-1)\hat{\nu }}}{2}=-d,0$$

However, if the absolute value of one of the eigenvalues is very small, then the overall dynamics of the system is characterized by rapid approach to a slow manifold, followed by a very slow approach toward equilibrium. Hence, we find a trade-off between requiring a small equilibrium fraction of stem cells while avoiding oscillations and the speed at which the system is able to recover from a perturbation.

The study of oscillations is an important part of feedback regulation. Damped oscillations have been observed in healthy hematopoiesis (Marciniak-Czochra and Stiehl, 2012). Amongst pathologies periodic oscillations are a characteristic feature of cyclical neutropenia (Bernard et al., 2003). Oscillatory behavior has also been identified in chronic and acute myeloid leukemia (Andersen and Mackey, 2001; Colijn and Mackey, 2005; Adimy et al., 2006). Moreover it was shown in Nakata et al. (2012) that in a three compartment model with feedback on the cell division rate, the destabilization of the positive equilibrium can lead to oscillations with a constant amplitude.

Going back to the requirements (*b* > 0) that guarantee the existence of a stable non-trivial steady state we note that they are independent of feedback inhibition on the division rate. Moreover for a fixed equilibrium division rate $\hat{v}$ the steady state population sizes are independent on the actual function *v*(*D*). The role of feedback on the division rate in the system lies instead in increasing the speed at which the system recovers from a perturbation and reducing the amplitude of oscillations if they happen to occur. This result is consistent with numerical simulations performed in Marciniak-Czochra et al. (2009), where it was observed that for short-time dynamics the coexistence of both regulatory mechanisms improves the efficiency of hematopoietic regeneration. Intuitively, oscillations occur when the number of differentiated cells is at equilibrium but the number of stem cell is not. If for example $S>Ŝ\;\text{and}\;D=\hat{D},$ then while the number of stem cells decreases toward its equilibrium value, the number of differentiated cells would grow. However, if there is feedback on the division rate, the difference between the rate of differentiated cell production and depletion 2(1 − *p*(*D*))*v*(*D*)*S* − *dD* would be smaller than in the absence of feedback $\left(2\left(1-p\left(D\right)\right)\hat{v}S-dD\right)$ and thus the maximum number of differentiated cells reached before the growth is reversed will not be as high. In the next sections we will present some numerical examples.

### Feedback inhibition using Hill equations

In this section we use Hill functions to model feedback inhibition equation (9):

(9) $$p\left(D\right)=p_{0}∕\left(1+gD^{m}\right),\;v\left(D\right)=v_{0}∕\left(1+hD^{n}\right)$$

Hill functions are widely used to describe ligand-receptor interactions (Alon, 2007), which makes them natural choices to model the actions of secreted feedback factors. Moreover they have been previously used to model the specific phenomena of cellular control for cell lineages in various tissues (Lander et al., 2009; Marciniak-Czochra et al., 2009; Chou et al., 2010; Bocharov et al., 2011; Zhang et al., 2012).

From expression equation (9) first note that the maximum self-renewal probability *p*_0_ must satisfy 0.5 < *p*_0_ ≤ 1. The value of *b* (defined in the previous section) in this case equals 1/(2*p*_0_). Hence the condition *b* > 0, which is necessary and sufficient to guarantee the existence of a stable steady state, is always satisfied.

Let us look now at the issue of oscillations near the steady state in relation to the equilibrium fraction of stem cells. In this case the discriminant of the eigenvalues equals:

(10) $$\Delta ={\left(\frac{d}{2p_{0}}\right)}^{2}-4\nu_{0}d\left(1-\frac{1}{2p_{0}}\right)$$

If we once again call $∊\equiv d∕\hat{v},$ then the condition Δ ≥ 0 can be rewritten as:

(11) $$∊>8p_{0}\left(2p_{0}-1\right)$$

If we require for example that the equilibrium fraction of stem cells is less than 10%, then ( < 0.111. Substituting this value into the previous equation we find that −0.0134 < *p*_0_ < 0.5134 and given that *p*_0_ > 0.5 we have:

(12) $$0.5<p_{0}<0.5134$$

**Figure 2:**
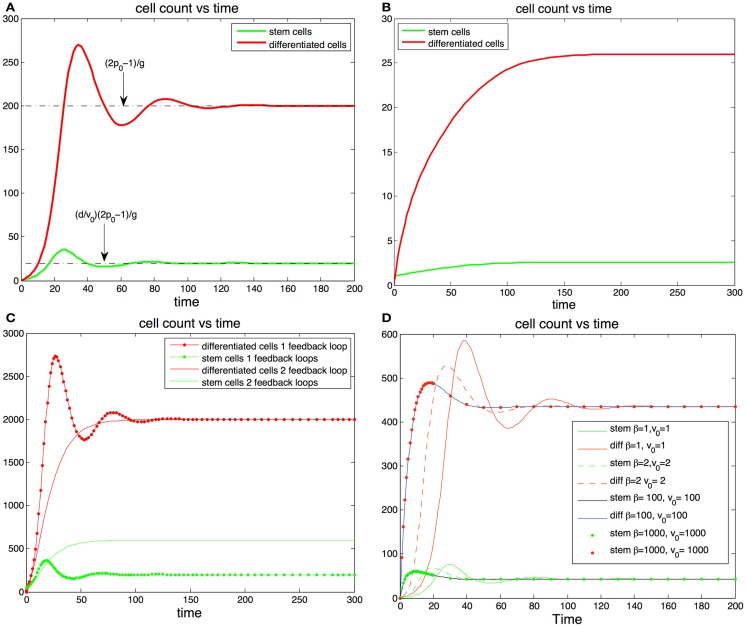
**(A,B)** Cell population with one feedback loop. **(A)** The trajectories oscillate toward steady state values (dotted line). Parameters, *p*_0_ = 0.6*, d* = 0.1*, g* = 0.001*, S*(0) = 1*, D*(0) = 0. **(B)** If there is only one feedback loop the maximum self-renewal probability must be very close to 0.5 to ensure that the trajectories approach the steady states monotonically. In this subfigure *d* and *g* are the same as in **(A)** but *p*_0_ = 0.513. **(C,D)** cell population with two feedback loops. **(C)** The steady state number of differentiated cells depends only *p*_0_ and *g* and is independent of feedback on the division rates. The steady state number of stem cells increases when the number of feedback loops increase from one to two. The addition of feedback in the division rate dampens or altogether eliminates the oscillations. **(D)** Fitting fixed steady state values of stem cells and differentiated cells values with different levels of feedback inhibition in the division rate. The stronger the feedback signal in the division rate the smoother the transition the equilibrium transition to equilibrium.

Hence, in a vicinity of the steady state, non-oscillatory trajectories that result in less than 10% of stem cells at homeostasis require that *p*_0_ lies within the small interval [0.5, 0.5134] (see Figure 2B). These findings suggest that the maximum self-renewal probability is very close to 0.5.

Interestingly a small value of *p*_0_ may have advantageous effects in the protection against cancer. Indeed the absence of feedback on differentiation leads to uncontrolled cell growth (Rodriguez-Brenes et al., 2011). Thus, having a small maximum self-renewal probability would result in a slower tumor growth rate in the event that feedback inhibition is lost. However, as we mentioned earlier this comes at the cost of reducing the speed of regeneration. In Figures 2A,B we track the trajectory of a cell population that has feedback on stem cell differentiation only (i.e., constant *v*(*D*)). In Figure 2B the fraction of stem cells is less than 10% and the maximum self-renewal probability is kept small (*p*_0_ = 0.513). Note how the system is able to recover from a severe perturbation (*D*(0) = 0) without presenting oscillations.

In Figures 2C,D we show results with feedback inhibition in both the self-renewal probability and the division rate of stem cells. As we discussed in the previous section, the addition of feedback on the division rate provides for smoother recoveries after a perturbation. Let us call β(*D*) = 1 + *hD^m^*, then *v*(*D*) = *v*_0_/β(*D*) and β(*D*) controls the strength of the inhibition signal. Clearly we can get a specific target division rate at equilibrium $\hat{v}$ with different combinations of the pair (*v*_0_, β(*D*)); the larger the magnitude of these quantities, the stronger the feedback in the division rate will be. In Figure 2D we plot different trajectories for the same target number of cells with different combinations of the pair (β, *v*_0_). Adding feedback inhibition on the division rate significantly dampens the magnitude of the oscillations and increases the speed at which the trajectories reach the steady states. The stronger the feedback signal the stronger the effect. Thus, even if feedback on the division rate is unnecessary to establish control, it promotes a faster and more stable recoveries in the system.

### Robustness

One of the negative consequences of oscillations may be the loss of the stem cell population which would result in the eventual extinction of the tissue. In this section we explore sufficient conditions that guarantee the survival of a population that starts at a critical level. In the ode model when a stable equilibrium exists it is easy to prove that zero is a repellent fix point. Hence the zero state cannot be reached from positive initial conditions. In practice this means that the stem cell population cannot hit zero as a result of a perturbation. Therefore to study extinction in the deterministic system we decide that extinction occurs when the number of stem cells falls below one (in the next section we present a stochastic formulation where complete extinction occurs). More precisely, we want to answer the following question: given a set value $\hat{D}$ and the initial critical conditions *S*(0) = 1 and *D*(0) = 0, can we find a parameter region that guarantees the survival of the population? From the eigenvalue analysis we found that in a vicinity of the steady state, the magnitude of the oscillations is determined by the discriminant in equation (10) and everything being equal, a greater value of *p*_0_ produces stronger oscillations. With this idea in mind we assume that given a choice of parameters *v*_0_, *d*, β that guarantee survival for a large upper bound self-renewal probability *p*_0_ = 0.9 and $g=\left(2p_{0}-1\right)∕\hat{D},$ then the same set of parameters guarantees survival for any other pair *p*_0_, *g*, that satisfies $\left(2p_{0}-1\right)∕g=\hat{D}$ and *p*_0_ < 0.9. Furthermore, the addition of the feedback on the replication rate increases the value of Ŝ and appears to dampen oscillations. Hence, we assume that any set of parameters that guarantee survival of the population with only one feedback loop should also guarantee survival when the two feedback loops are in place.

**Figure 3:**
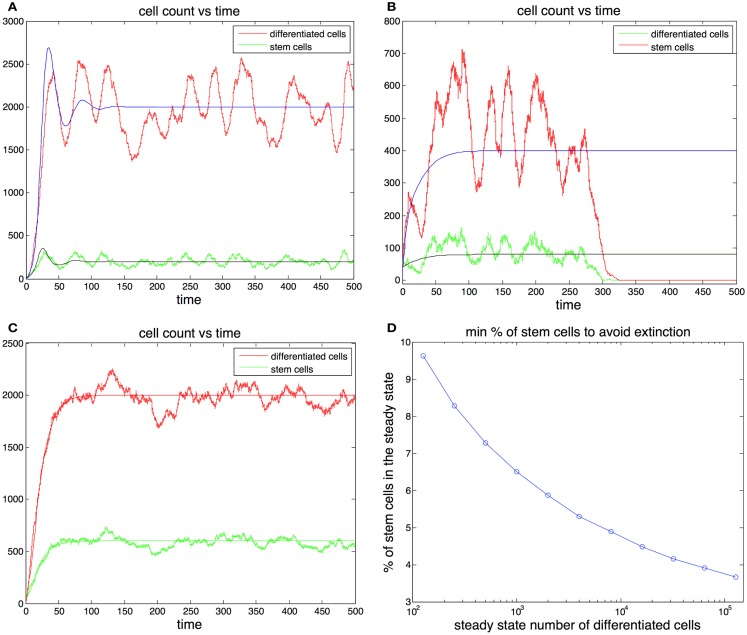
**(A,B)** Cell population with one feedback loop. The stochastic simulation is shown in red for differentiated cells and green for stem cells. The ode is shown in blue for differentiated cells and black for stem cells. Parameters in **(A)** *p*_0_ = 0.6, *d* = 0.1*, g* = 0.0001*, h* = 0*, S*(0) = 10*, D*(0) = 0. Parameters in **(B)** *p*_0_ = 0.52*, d* = 0.2*, g* = 0.0001*, h* = 0*, S*(0) = 40*, D*(0) = 0. **(C)** Cell population with two feedback loops. Feedback in the division rate dampens oscillations. Parameters are the same as in **(A)** with the exception *h* = 0.001. **(D)** Sufficient conditions for the survival of the population in the ode model. Let us call the curve in the graph *y*($\overset{⌣}{D}$). Then for any set of parameters that satisfy (2*p*^0^ − 1)/*g* = $\overset{⌣}{D}$, *p*^0^ (0.5, 0.9) and the steady state fraction of stem cells *f* $\underline{\underline{>}}$*y($\overset{⌣}{D}$)*, the initial conditions *S*(0) = 1, *D*(0) = 0 guarantee the survival of the population. For example, for all $\overset{⌣}{D}$ $\underline{\underline{>}}$ 10^3^ if *p*_0_ $\underline{\underline{<}}$ 0.9 and the steady state fraction of stem cells *f* $\underline{\underline{>}}$ 0.064 survival is guaranteed for any level of feedback on the division rate. (These conditions are sufficient but not necessary.)

The previous considerations reduce our search to pairs (*d*, *v*_0_) that guarantee survival, given the initial conditions $\left(p_{0}=0.9,\;g=0.8∕\hat{D},\;\beta =1\right).$ Finally we note that the amplitude of the oscillations depends on the ratio *d*/*v*_0_ and not on the actual magnitude of *d* and *v*_0_ so we only need to test different values for this ratio. Since this ratio is closely related to the percentage of stem cells by the equality $Ŝ=d∕v_{0}\hat{D},$ then the results can be presented in terms of the steady state percentage of stem cells (Figure 3D).

The analysis performed here indicates that in the ode model there are ample parameter regimes that guarantee the survival of the population while maintaining a small stem cell load. In general the greater $\hat{D}$ is the smaller the equilibrium fraction of stem cells may be to guarantee survival. Moreover in this analysis the system was required to rebound from very extreme initial conditions (*S*(0) = 1). In practice most injuries that are able to heal would rarely include populations that are reduced to a single cell. Furthermore, as we found earlier the addition of feedback in the division rate and the reduction of the maximum self-renewal probability *p*_0_ further increase the stability of the system.

### Stochastic model

We are also interested in studying the effects of stochastic fluctuations in the model. With this aim in we implement the following algorithm using Gillespie’s Method (Gillespie, 1977).

**Algorithm:**

Assume that at time *t*, the system is described by the pair (*S*(*t*), *D*(*t*)), and *r*1, *r*2, and *r*3 are random numbers uniformly distributed in [0, 1].

1. Set *p*(*t*) = *p*_0_/(1 + *gD*) and *v*(*t*) = *v*_0_/(1 + *hD*).
2. Compute *a* = *v*(*t*)*S*(*t*) + *dD*(*t*).
3. Set the new time *t*′ = *t* − 1/*a* · log(*r*1).
4. If *a* · *r*2 < *dD*(*t*), the next event is cell death of a differentiated cell, hence make *D*(*t*′) = *D*(*t*) − 1.
5. If *a* · *r*2 > *dD*(*t*), the next event is stem cell division. If *r*3 < *p*(*t*) the cell divides into two stem cells, hence make *S*(*t*′) = *S*(*t*) + 1. If *r*3 > *p*(*t*) the cell divided into two differentiated cells, hence make *S*(*t*′) = *S*(*t*) − 1 and *D*(*t*′) = *D*(*t*) + 2.

In Figures 3A,B we plot two stochastic simulations with only one feedback loop together with the corresponding ode formulations. Note that in Figure 3B the simulation ends with the extinction of the cell population, even though the ode model does not go extinct. In general the extinction of the cell population is a more likely event when the steady state number of stem cells is small, given that random deviations from the mean can bring the number of stem cells to zero. The addition of a second feedback loop (Figure 3C) increases the stability and reduces the variance in the number of cells. A realization of the algorithm is a random walk that represents the distribution of the master equation, and which captures the stochastic fluctuations typically observed in systems with a small number of agents. As the number of cells increases, the fluctuations in the number of cells decrease and the thus the stochastic realizations increasingly resemble the corresponding trajectories produced by the ode (Gillespie, 1977).

There are two more things worthy of being noted. First the occurrence of random fluctuations makes the stochastic model even more sensitive to oscillations. Second in a stochastic setting an injury that severely depletes the number of cells is not guaranteed to be able to rebound and there may be a significant chance of extinction. These observations suggest that the control mechanism considered so far is not well suited for systems that rely on a critically small number of stem cells, such as the colon lining which may rely on as little as four stem cells per crypt (Marshman et al., 2002). Instead it is better suited to deal with systems with a large number of cells such as blood (Shizuru et al., 2005). Moreover the use of mass action equations assumes a well mixed system, which is a reasonable assumption for non-solid tissues. In the next section we will discuss the effects of adding spatial interactions to the model.

### Spatial model

#### The spatial effects

**Figure 4:**
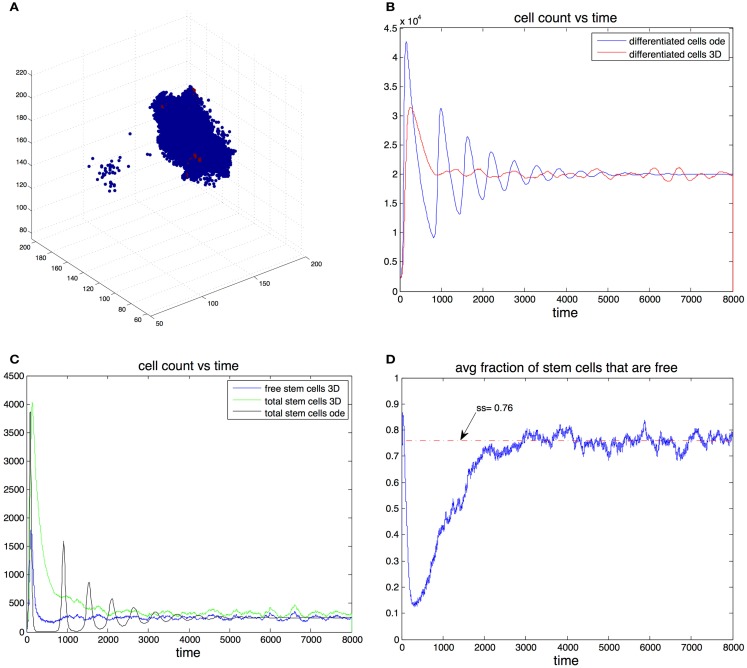
**(A)** Example of the spatial arrangement of the cell population in three dimensions. Differentiated cells are shown in blue and stem cells in red. **(B)** Cell count of differentiated cells vs. time. The blue line was computed using the ode model, the red line is the expected cell count in the spatial model. **(C)** Cell count of stem cells. Results form the ode (black) and expected cell count in spatial-dimensional model (green). The expected number of cells is in the spatial model is shown in blue. **(D)** Expected fraction of stem cells that are free in the three-dimensional model. Parameters in all figures are: *p*_0_ = 0.7, *v*_0_ = 0.2, *g* = 2 × 10^5^, β = 1, and *d* = 0.0025.

In this section we consider cell dynamics in three dimensions. We assume that cells are restricted to a three-dimensional rectangular lattice of *n*_I_ × *n*_J_ × *n*_K_ points. A lattice point can host at most one cell at any time. The position of each cell can be determined by its coordinates in the lattice (*i*, *j*, *k*), *i* = 1, …, *n*_I_, *j* = 1, …, *n*_J_, and *k* = 1, …, *n*_K_. Following the rules of the previous sections, stem cells divide either into two stem cells or two differentiated cells. For a cell to divide, there must be a free lattice point adjacent to it. If the cell divides, then one offspring remains in the position occupied by the parent cell and the other occupies a position adjacent to the cell. There are cases in which a cell that is able to divide has more than one free adjacent lattice point that may be occupied by one of its two offspring. In this case we choose the site randomly, with each adjacent free lattice point having the same probability of hosting one of the two daughter cells. The events are chosen using the stochastic simulation algorithm (described above) modified to take into account the spatial rules. A graphical representation of the spatial arrangement of the three-dimensional cell population is given in Figure 4A.

We found that adding space to the system results in smoother transitions from perturbed to equilibrium configurations. Compared to the non-spatial system, if oscillations are observed, the amplitudes are significantly reduced, which in turn results in much fewer instances that end with the stochastic extinction of the cell population. This behavior is exemplified by Figures 4B,C. Here we picked parameter regime (*p*_0_ = 0.7, *v*_0_ = 0.2, *g* = 2 × 10^−5^, β = 1, *d* = 0.0025) that produces oscillations in the non-spatial model. The initial conditions are $\left(S\left(0\right),D\left(0\right)\right)=0.1\left(Ŝ,\hat{D}\right),$ where $\left(Ŝ,\hat{D}\right)$ are the steady state values from the ode model. With this initial conditions the number of stem cells in the ode model falls below one, which in practice means that the population goes extinct. Furthermore we performed 100 independent simulations using the stochastic non-spatial model and every one of them resulted in the extinction of the cell population. In contrast not one of 30 simulations using the spatial model resulted in extinction.

In the non-spatial model the steady state fraction of stem cells is:

(13) $$\frac{\hat{S}}{\hat{S}+\hat{D}}=\frac{d\beta /\nu_{0}}{d\beta /\nu_{0}+1}$$

In the spatial model this quantity gives the steady state percentage of free stem cells – cells that have free space in an adjacent position in the grid and are thus able to divide. This means that for a given set of parameters, the equilibrium number of stem cells in the spatial model is greater than the equilibrium number in the non-spatial model. For example in Figure 4C the steady state fraction of stem cells in the ode model was approximately 0.0123 (as predicted by the formulas). In the three-dimensional model the expected steady state fraction of cells was approximately 0.0165, an increase of about 32% from the deterministic model’s prediction.

The mechanism by which the spatial model is able to achieve a greater stability can be inferred by looking at Figures 4C,D. At the start of the simulation the number of differentiated cells is only 10% of the steady state value. Therefore the probability of differentiation is small and stem cells have a high probability of dividing into two stem cells. Once the number of differentiated cells is above $\hat{D},$ differentiation becomes the more likely event and in the ode model one sees a steep reduction in the number of stem cells that leads to extinction. In the spatial model however, the rapid growth phase of stem cells means the fraction of free cells is reduced as most stem cells are trapped by other stem cells. Only these free stem cells are able to divide, slowing down the speed at which stem cells are depleted. It is important to note that the spatial effects in this model act locally by reducing the space available for cell division, their strength thus depends on the degree of the graph. As the graph degree increases the spatial effects become weaker until eventually the mass action dynamics are fully recovered.

In a spatial setting the stem cell niche concept (Morrison et al., 1997; Simons and Clevers, 2011) might also play a role in promoting stability. If the amount of space in the niche were limited, this would place a cap in the maximum number of stem cells, which could in turn decrease the overshooting of the stem cell number observed during oscillations. Exactly how the explicit modeling of these microenvironments might affect the performance of the regulatory mechanisms investigated here should be the subject of future research.

## Discussion

In this article we studied the cell dynamics that arise from feedback inhibition in the self-renewal probability of stem cells and their division rate. We found that by itself feedback on the probability of self-renewal is sufficient to establish control and uniquely determines the equilibrium number of differentiated cells. The equilibrium fraction of stem cells on the other hand depends solely on the ratio of the death rate and the rate of stem cell division.

In the process of recovering after an injury this control mechanism may produce oscillations in the number of cells, a behavior that may be dangerous and of no obvious biological value. Near equilibrium oscillations are more likely to occur when the steady state fraction of stem cells is small. If this is the case, avoiding oscillations is still always possible, but it comes at the price of reducing the speed at which the cell populations recover from a perturbation. If feedback inhibition follows a Hill equation, avoiding oscillations while maintaining a small stem cell load requires that the maximum self-renewal probability be only slightly larger than one-half. Feedback inhibition on the stem cell division rate does not affect the steady state values of either stem cells or differentiated cells, but it reduces the amplitude of oscillations if they happen to occur. Furthermore it can increase the speed of recovery after an injury, altogether promoting faster and more stable recoveries of the cell population.

On occasions, extreme oscillations may result in the extinction of the entire population. However, we find that there are ample parameter regimes in which this doesn’t occur, even while the system is recovering from severe initial conditions. We found that the larger the equilibrium number of differentiated cells, the smaller the equilibrium fraction of stem cells may be while still avoiding extinction. Due to fluctuations, in a stochastic setting the danger of extinction through oscillations is greater. This suggests that the mass action model is only well suited as a quantitative tool for tissues where the steady state number of stem cells is not critically small.

We also explored how spatial interactions affect the cell dynamics in a stochastic setting. We found that spatial effects greatly reduce oscillations and the chances of random extinction, providing smoother transitions from a perturbed state to equilibrium. This increase in stability is achieved by reducing the number of stem cells that are capable of division at a given time. When recovering from an injury the rapid expansion of the stem cell population traps some of the stem cells, making them incapable of cell division. Hence, when the steady state number of differentiated cells is reached, a significant fraction of stem cells cannot divide. This reduces any possible further increase in the number of differentiated cells causing the magnitude of any oscillation to decrease as well.

The models of hierarchical cell populations studied here are relevant to both healthy and cancerous tissues. In Rodriguez-Brenes et al. (2011) we showed how cancer could develop from healthy hierarchical tissues by a unique sequence of phenotypic transitions, which gradually lead to a complete escape from regulation in stem-cell-driven tumors. Moreover, we compared the resulting tumor growth patterns with existing tumor growth data and saw that in many instances, the regulatory mechanisms of healthy tissues continue to operate to a degree in tumors. This underlines the importance to cancer biology of studying the principles of tissue regulation. Another example of the relation between tissue regulation and the process of carcinogenesis is found in Stiehl and Marciniak-Czochra (2012).

One important result for the cancerous transformation found in Rodriguez-Brenes et al. (2011) is that the negative feedback loops controlling the differentiation decisions must be the first to be inactivated. The breakage of the division control loops must happen at a later stage of carcinogenesis. Here we reevaluate this finding from a different perspective. In order to achieve the deregulation of divisions and rapid growth, cancerous cells must first acquire a mutation deactivating the differentiation control. Otherwise, the tissue may become unstable and enter stochastic fluctuations preventing steady growth. Therefore, a one-step transformation from healthy tissue to a tissue with no division control mechanism is highly unlikely. This can be viewed asa protection mechanism that organs put in the way of cancerous transformations, making the transition to cancer more difficult and statistically delaying the onset of cancer (for related ideas, see also (Komarova and Cheng, 2006)).

The optimization task for healthy hierarchical tissues is to provide stable maintenance and a quick and reliable recovery from injuries. Over time, tissues have evolved (at least partially) to reach these objectives. In contrast, therapeutic approaches often pursue the opposite tasks: the destabilization of cancerous tissue, increasing the chance of stochastic extinction (say, after a course of chemotherapy or surgery) and the slowing down of tumor growth. Our models show what parameters (and to what degree) are responsible for stability and growth. Understanding how various parameters contribute to cell population growth and stability can lead to novel ideas for cancer treatments, where one could target factors leading to growth retardation or destabilization.

## Conflict of Interest Statement

The authors declare that the research was conducted in the absence of any commercial or financial relationships that could be construed as a potential conflict of interest.
